# Monothetic Analysis and Response Surface Methodology Optimization of Calcium Alginate Microcapsules Characteristics

**DOI:** 10.3390/polym14040709

**Published:** 2022-02-12

**Authors:** Joshua Anani, Hussien Noby, Abdelrahman Zkria, Tsuyoshi Yoshitake, Marwa ElKady

**Affiliations:** 1 Chemical and Petrochemicals Engineering, Egypt-Japan University of Science and Technology, Alexandria 21934, Egypt; joshua.anani@ejust.edu.eg (J.A.); hussien.badry@ejust.edu.eg (H.N.); 2 Materials Engineering and Design, Faculty of Energy Engineering, Aswan University, Aswan 81528, Egypt; 3 Department of Applied Science for Electronics and Materials, Kyushu University, Kasuga 816-8580, Japan; abdelrahman_zkria@kyudai.jp (A.Z.); yoshitake.tsuyoshi.913@m.kyushu-u.ac.jp (T.Y.); 4 Department of Physics, Faculty of Science, Aswan University, Aswan 81528, Egypt; 5 Fabrication Technology Department, Advanced Technology and New Materials Research Institute (ATNMRI), City of Scientific Research and Technology Applications, Alexandria 21934, Egypt

**Keywords:** biopolymer, electrospraying, calcium alginate, surface responses methodology, microcapsules

## Abstract

Owing to bio-polymer’s low-cost, environmental friendliness and mechanically stable nature, calcium alginate microcapsules have attracted much interest for their applications in numerous fields. Among the common production methods, the Electrospraying technique has shown a great potential due to smaller shape capsule production and ease of control of independent affecting parameters. Although one factor at a time (OFAT) can predict the trends of parameter effect on size and sphericity, it is inefficient in explaining the complex parameter interaction of the electrospray process. In the current study, the effects of the main parameters affecting on size and sphericity of the microcapsules using OFAT were optimized to attain calcium alginate microcapsules with an average diameter below 100 µm. Furthermore, we propose a statistical model employing the Surface Responses Methodology (RSM) and Central Composite Design (CDD) to generate a quadratic order linear regression model for the microcapsule diameter and sphericity coefficient. Experimentally, microcapsules with a size of 92.586 µm and sphericity coefficient of 0.771 were predicted and obtained from an alginate concentration of 2.013 *w*/*v*, with a flowrate of 0.560 mL/h, a needle size of 27 G and a 2.024 *w*/*v* calcium chloride concentration as optimum parameters. The optimization processes were successfully aligned towards formation of the spherical microcapsules with smaller average diameter of less than 100 µm, owing to the applied high voltage that reached up to 21 kV.

## 1. Introduction

Alginate is a broad definitive term for a group of naturally occurring unbranched polysaccharides consisting of 1.4 linked β-D-mannuronic acid, a C-5 epimer and an L-guluronic acid. It is abundant in nature and mainly produced from seaweed, bacteria and algae, with around 30,000 metric tons of annual production [1]. Due to their abundance and versatility in application, alginates have seen significant applications as beads [2], fibers [3] and capsules of micro and nano sizes [4] in diverse fields ranging from biological and medical to chemical and mechanical use. In delivery systems, alginates have found an abundance in application due to their environmentally friendly nature, solubility in water, excellent biodegradability and compatibility [5]. Spherical hydrogel capsules and beads of alginates have been used widely for drugs, enzymes and cell delivery in the medical and biomedical sciences. Hydrogel Micro and nano alginate capsules have also gained recent popularity as an encapsulating material for anti-corrosion materials [6,7]. Alginate beads have a wide range of applications as encapsulating anti-corrosion materials due to their ease of use, low cost, lack of toxicity, mechanical stability and resilience to acidic and basic environments [8].

The primary alginate micro and nanocapsules production techniques are extrusion and emulsification/gelation. External extrusion techniques are the most popular, due to their simplicity of application and particle size distribution control [9]. However, the internal emulsification/gelation technique has the advantage of producing smaller capsule sizes [10,11]. A less common technique for producing alginate capsules is the spray drying technique. Typically, a mixture of alginate solution and organic reagents is atomized by hot airflow. The shortcoming of this technique is the product loss to the drying chamber walls and the loss of finer particles (<2 µm) as exhaust air [12]. The aforementioned techniques are essential to producing alginate capsules as carriers for anti-corrosion agents in self-healing coatings. Typically, alginates are available in commercial quantities as salts of sodium alginic acid and gum, although other salts of alginates are available.

The general preparation of alginate microcapsules involves ionic gelation of alginate, which comprises simple diffusion and crosslinking process with divalent cations, typically Ca^2+^ [13]. Calcium salts are used for crosslinking as they are economical, safe and easy to use. The alginate gelation occurs when the divalent cation binds itself to two carboxyl groups of an alginate molecule, as thoroughly documented and explained by the egg-box model [14]. The size, sphericity and shape of the capsules play a crucial role in the field of application of the polymer. Smaller-sized and spherical microcapsules are crucial as encapsulating materials for anti-corrosion materials in self-healing applications. The capsule’s resistance to shear and compressive pressures increases as its size decreases [15]. Excellent shear and compressive forces are essential to controlled release in self-healing for anti-corrosion. Smaller capsules can provide significant solid-liquid interface gaps in a given volume, facilitating substrate and product mass transfer [16,17] Alginate sphericity also has a direct correlation with chemical and mechanical stability. Spherical capsules have shown remarkable and more effective gel strength than non-spherical ones. Premature encapsulant burst and release are also due to tears, cracks and breakages of non-spherical capsules [18].

The extrusion technique for alginate microcapsules involves forcing alginate solutions as droplets into the crosslinker through a nozzle. Extrusion is usually associated with larger alginate beads, usually of sizes >1000 µm. However, the electrospray/electrodynamic atomization technique can produce smaller-sized microcapsules [19].

Electrospraying/electrodynamic atomization has seen recent advancements in the production of microcapsules in a more controlled manner. This technique uses electrostatic forces to overcome polymer surface tension and viscosity, extruding the fluid through a needle tip. In the presence of an electric potential, the extruding polymer, a conical-shaped micro or milli-sized droplet, accelerates as streams towards a crosslinking solution at the bottom where it is collected [20]. Various factors affect the size and sphericity production of the microcapsule; such as needle size, alginate concentration, electric potential and flowrate [21].

The emulsification technique is a small size-oriented technique for alginate microcapsule production [22]. In this technique, alginate solution is mixed with a suspension of insoluble calcium salts for gelation with a surfactant in an oil phase. An acid or a base adjusted the pH of the solution to guarantee complete gelation. Homogenization, stirring and sonication all influence the size reduction of the alginate microcapsule.

There have been several (OFAT) analyses of the effects of different variables on particle size and sphericity. However, OFAT analyses are inconclusive, and cannot explain the behaviors of the independent variables at some range of studies. Calcium alginate microcapsules have been optimized using response surface methodology [19,21] in prior studies. However, the range of study for the respective parameters was limited, especially for flowrate and voltage.

Herein, this study investigated OFAT experiments for the independent parameters at a wider range of high voltage and flowrate, showing the inconsistencies of the OFAT. Accordingly, the introduction of a linear regression model and an ANOVA study were investigated to explain the parameter interactions and how they affect size and sphericity as an attempt to formulate microcapsules with smallest average diameter below 100 µm.

## 2. Materials and Methods

### 2.1. Materials

Low viscosity (1% *W*/*V*, 5.5+/−2.0 cps) sodium alginate(C_6_H_9_NaO_7_) was purchased from Advent ChemBio PVT. Ltd, India. Calcium chloride 2-hydrate (CaCl_2_.2H_2_O) salt was purchased from AppliChem GmbH, Germany.

### 2.2. Synthesis of Calcium Alginates Using Electrospray Technique

**Figure 1:**
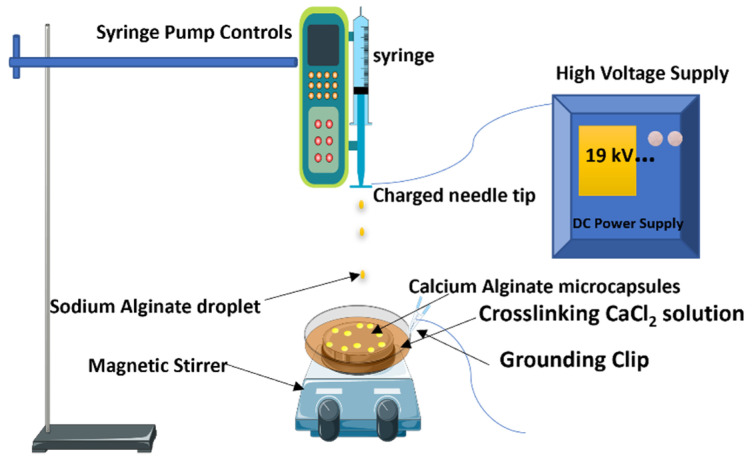
Elecrospray System Setup.

Various Sodium alginate solutions were made by dissolving sodium alginates in distilled water with the aid of a high-speed homogenizer. The solutions were left overnight for complete degassing. Each solution was poured into a 12 mL syringe with a blunted stainless needle of varying diameters. The filled syringes were inserted into the electrospray device’s pump, with the needle tip kept at a consistent distance (8 cm) from the collecting beaker. The sodium alginate polymer solution was supplied at varying flow rates into a beaker containing the crosslinking calcium chloride solution. A varying high voltage was applied to the tip of the needle. The calcium chloride solution was placed on a stirrer at a constant rpm of 150, and grounded using crocodile connector clips. The microcapsules collected into the crosslinker were washed thoroughly after allowing it to harden for 2 h. The blank microcapsules were dried in a vacuum oven at 50 °C overnight. Figure 1 is a representation of the electrospray process.

### 2.3. Microcapsule Size Distribution Determination

The different microcapsules produced by the electrospray technique were measured and analyzed using SEM (JEOL JSM-6390. Approximately 25 samples of the alginate microcapsules were selected and analyzed for each parameter combination and variation. The average minimum and maximum diameter were calculated from randomized samples using ImageJ software [19].

### 2.4. Sphericity Coefficient Determination

Different sphericity indicators have been used to determine the shapes of microcapsules and other minuscule structures [18,19].

For this study, the sphericity coefficient is preferred for calculating the average sphericity of the capsules for each parameter variation. It is calculated as illustrated in Equation (1).

(1) $$\mathrm{Sphericity}\;\mathrm{coefficient}=\frac{D_{L}}{D_{F}}\;$$

D_L_ is the lateral diameter and D_F_ is the mean frontal diameter of the capsule at varying positions. Sphericity of 1 indicates a perfect sphere, while sphericity approaching infinity indicates elongation, such as fibers.

Overall, the average sphericity coefficient was calculated from 25 random samples for each parameter variation run.

### 2.5. Experimental Size and Sphericity Optimization

The size and sphericity of the electrosprayed microcapsules were optimized with Design-Expert software (Stat-Ease, version 13.0.0) using a response surface methodology (RSM) and a central composite design (CDD). Rather than making a monothetic one-factor-at-a-time (OFAT) observation, the RSM employs statistical and mathematical approaches to study the interrelationship between independent factors and the desired response. After inputting the respective minimum and maximum independent factors from the responses collected from the diverse experimental runs generated by the software, a linear regression model was generated, employing quadratic process orders for subsequent response prediction. The equation for the quadratic model is illustrated in Equation (2)

(2) $$Y=\beta_{o}+\overset{K}{\underset{a=1}{\sum }}\beta_{a}X_{a}+\overset{K}{\underset{a=1}{\sum }}\beta_{\mathrm{aa}}X_{a}^{2}+\overset{K-1}{\underset{a=1}{\sum }}\overset{K}{\underset{b=a+1}{\sum }}\beta_{\mathrm{ab}}X_{a}X_{b}+\varepsilon$$

where Y is the dependent variable; X_a_ and X_b_ are the independent variables; β_o_ is the overall average response constant/intercept; β_a_ is the coefficient of the linear regression model on a linear level; β_aa_ is the effect of squaring the linear coefficient; β_ab_ is the effect of the interrelationship between the coefficients of the linear regression; and ε accounts for the error in the model.

Overall, the model was created with a single full block, resulting in 50 trials, calculated from the CCD model using Equation (3)

(3) $$N=2^{K}+2K+n$$

where K is the independent variables studied and *n* is the center points. The factor and response summary tables are stated in Table 1 and Table 2.

The ANOVA (Analysis of Variance), model regression, optimization, numerical and graphical assessment of the bead size and sphericity were studied.

### 2.6. Model Validation

Post-analysis model confirmation was done for the predicted optimized microcapsule diameter and sphericity conditions. Greater priority was given to the microcapsule size, at the expense of sphericity. The microcapsule diameter was set to a minimum with a 5-star importance level in the prediction. The minimum sphericity coefficient was set at 0.7, and the maximum at 0.99. The desired outcome was the smallest possible diameter less than 100 µm, with appreciable sphericity.

### 2.7. Optimization

The optimization for the microcapsule diameter and sphericity was conducted using Design-Expert Software. The microcapsule diameter received more attention compared to the sphericity. The independent variables were given equal significance in the optimization process.

## 3. Results

### 3.1. OFAT Analysis of Independent Parameters

In order to fabricate sodium alginate microcapsules with smallest diameter and acceptable degree of sphericity, the effect of fabrication parameters on capsule size and sphericity were investigated experimentally using an OFAT analysis.

#### 3.1.1. Effect of Sodium Alginate Concentration

**Figure 2:**
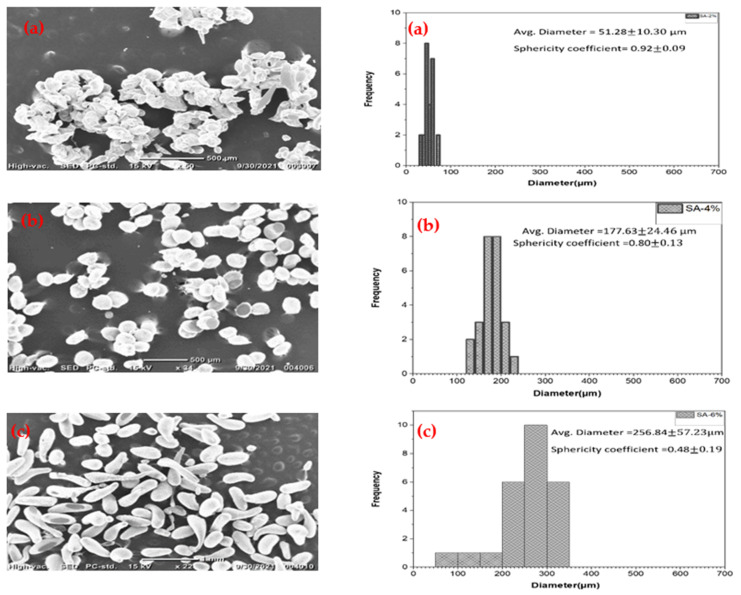
Monothetic analysis, SEM and distribution of 25 randomly selected Calcium alginate Microcapsules with varying Sodium Alginate concentrations (**a**) 2% *w*/*v*; (**b**) 4% *w*/*v*; (**c**) 6% *w/v*.

There was an increase in the particle size as the concentration of electrosprayed sodium alginate solution increased from 2% to 6% *w*/*v*, as denoted in Figure 2a. An increase in concentration leads to an increase in viscosity, and thus the extrusion of thicker fluid through the nozzle of the needle [23,24]. Figure 2 shows the distribution of microcapsule size with varying alginate concentrations. The 6% *w*/*v* microcapsules produced from the electrospray technique were elongated and misshapen due to increased surface tension at the needle-droplet surface, and as such, complexity in breaking off the viscous fluid at the needle tip [18,19]. Alginate sphericity decreased with increasing concentration. The higher viscosity of the solution could cause deformation of the microcapsule as it is electrosprayed into the crosslinking solution. Two forces act on an electrosprayed microcapsule as it moves from the tip of the needle into the crosslinker: the viscous forces and drag forces. The viscous force of the fluid seeks to maintain the microcapsule’s form, while the drag force seeks to disrupt it [18]. As such, a very low concentration of the sodium alginate solutions (below 2% *w*/*v*) that formed microcapsules had weaker walls, which were disrupted either in the crosslinker or during drying. On the other hand, the higher concentration alginate solution formed a sperm-like shape–a result also observed in another study [25], as also seen in Figure 2c. However, the interaction of other parameters, such as bigger needle size, could lead to spherical microcapsules at higher alginate concentrations.

#### 3.1.2. Effect of Voltage

The effect of applied voltage at the electrospraying process is a critical parameter in the microcapsule size variation. During electrospraying, the surface tension of the sodium alginate polyelectrolyte solution becomes equal to the Coulomb repulsion forces at the extruded jet point when pushed through a needle with an electric voltage [26]. The fluid at the needle’s tip creates a cone shape, referred to as the Taylor cone. Tiny droplets are sprayed spontaneously from the tip of the Taylor cone into a counter electrode when the electric field strength is greater than the balancing point. The development of a Taylor cone is critical for generating microcapsules [27], and heavily depends on operating parameters such as voltage and feed rate. A droplet on the tip of a needle develops, until it is heavy enough to escape the surface tension of the needle-droplet interface in the absence of an electric field, using the effect of gravity and the flow rate. At a higher voltage (>21 kV), it was investigated that due to hydrodynamic instabilities, a narrow jet from the tip of the cone splits into multiple jets, resulting in unstable stream formation, fibers and non-uniformed capsule formation. The capsule sizes formed from lower voltages (<13 kV) were too large for this investigation. Very spherical microcapsules were formed at a lower voltage.

#### 3.1.3. Effect of Flowrate

**Figure 3:**
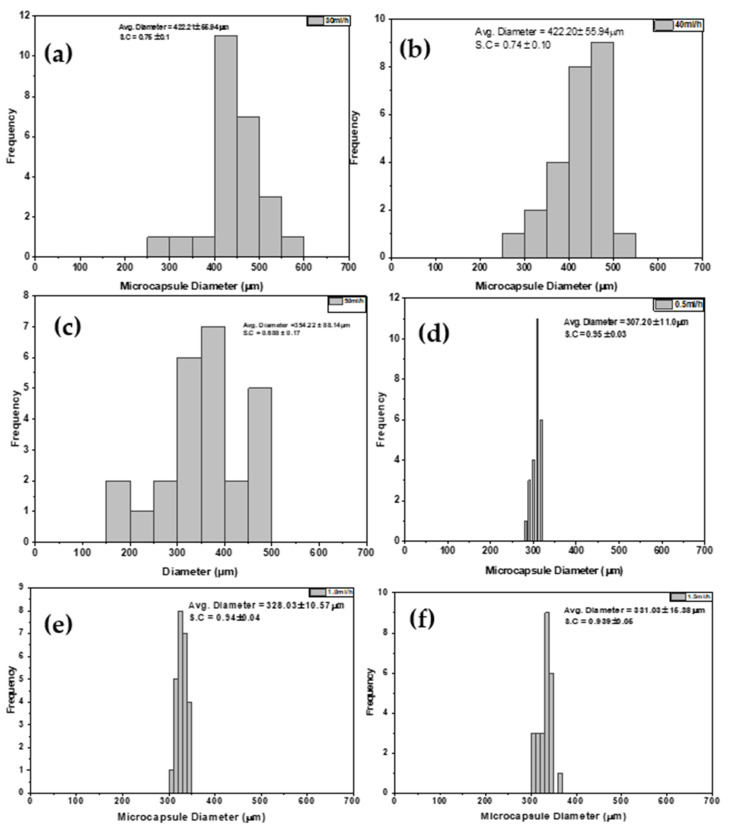
Microcapsule size distribution of calcium alginates electrosprayed at high (**a**–**c**) and low flowrates (**d**–**f**).

Flowrate typically has an inverse relationship with microcapsule size and sphericity. In Figure 3d–f, lower flowrates formed smaller-sized and better spherical microcapsules as compared to higher flowrates. The capsule size increases gradually and sphericity decreases gradually with increasing flowrates. However, the trend changes at higher flowrates in Figure 3a–c, as size starts to decrease while sphericity continues to reduce. This observation could be due to the interaction of parameters at high flowrates, causing size changes. A high flow rate increases the size of the droplets at the nozzle, lowering the surface charge density and increasing the capsule diameter [28]. When the flow rate increases, the sphericity decreases significantly, as there is no time to properly construct a Taylor cone at the tip of the needle. As a result, tear-shaped, pear-shaped and elliptical capsules develop. At very high flowrates, inconsistencies in trends were observed.

#### 3.1.4. Effect of Calcium Chloride Concentration

**Figure 4:**
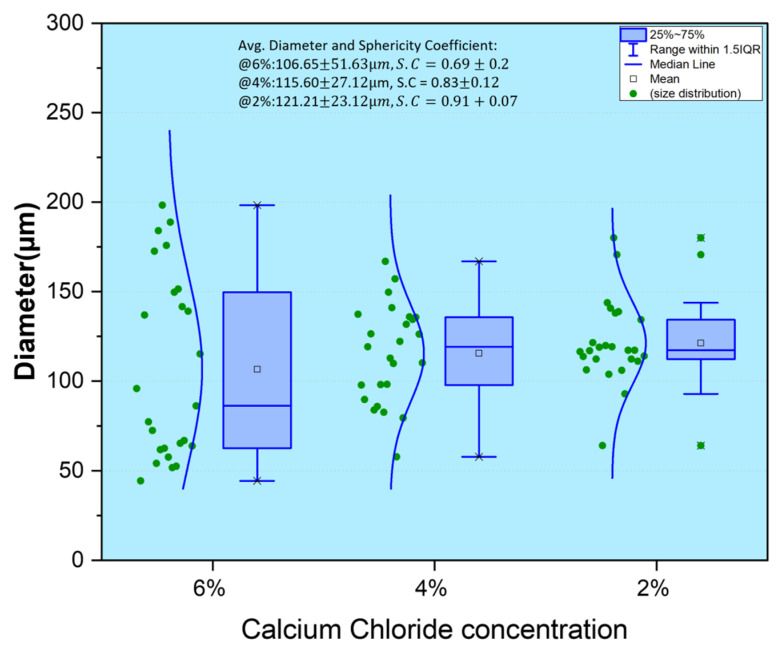
Box and Whiskers plot of the monothetic analysis of 25 randomly selected calcium alginate microcapsules crosslinked with varying concentrations of Calcium Chloride.

Calcium chloride concentration has an inverse effect on the particle sizes of formulated sodium alginate microcapsules. The higher the calcium chloride concentration, the higher the shrinkage of the microcapsules produced, and therefore, the smaller the particle size [24]. However, the sphericity coefficient reduces with increasing concentration, as the microcapsules are hardened disproportionately, leading to smaller yet non-spherical particles. Microcapsules made from <2% *w*/*v* CaCl_2_ solution failed to harden, had weak walls and imploded, owing to inadequate crosslinker and hardening salt concentrations to produce stable microcapsules. From Figure 4, the 6% *w*/*v* calcium chloride microcapsules showed a smaller average particle size, with sporadic distribution indicating polydispersity and an overall poor average sphericity coefficient. The 2% *w*/*v* showed a larger average particle size fractionally, but with an impressive sphericity coefficient and a better-monodispersed range. Most of the 2% microcapsules were similar to the 4% *w*/*v* microcapsules, except for some outliers. The outliers of the 2% *w*/*v* microcapsules are few enough to be included in the margin of error. The sphericity coefficient also favored the 2% *w*/*v* concentration microcapsules. The effect of calcium chloride as an independent parameter had little significance on the overall bead diameter compared to other strong parameters, such as voltage, needle size and alginate concentration.

#### 3.1.5. Effect of Needle Size

**Figure 5:**
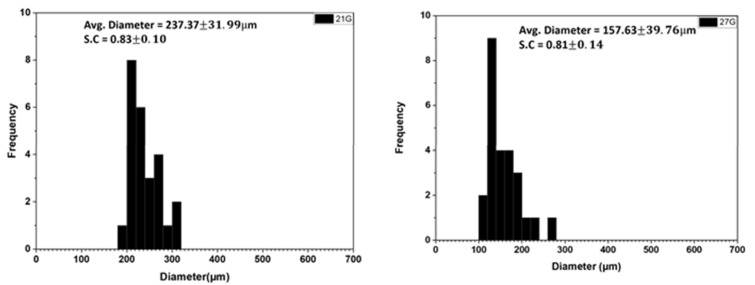
Monothetic Analysis of 25 random calcium alginate microcapsules electrosprayed through varying needle diameter.

Needle size has a direct relationship with formulated calcium alginate microcapsule size. The smaller the orifice of extrusion, the smaller the microcapsule extruded [29]. As illustrated in Figure 5, two-needle sizes (21 and 27 G) were studied with all other parameters constant. The 27 G needle performed better than the 21 G needle, with a smaller particle size with a mean diameter of 157.63 µm as compared to the 27 G needle, which had a size of 237.37 µm. Overall, both showed a good distribution. Needle size is the most important parameter in diameter control [19]. The orifice of the needle controls the amount of fluid that flows to the surface of the needle. Therefore, the amount of alginate at the tip of the needle is controlled by needle size. The droplet at the tip is then acted on by voltage to reduce the size. The extent of reduction in diameter of the alginate droplet at the tip by voltage is dependent on the size of the polymer droplet supplied to the tip by the needle orifice.

Generally, according to all previously studied parameters affecting on microcapsules formulation, the investigations showed normal behaviors for most studied parameters, although some deviations from the general trends of other studied variables on capsule size and sphericity were observed, depicting that the variable interactivity influenced capsule size and sphericity.

### 3.2. ANOVA and Model Generation

The OFAT analysis of the effects of the various parameters showed varying inconsistencies, which can only be explained by analyzing the variable parameter interactions and the degree of their effects on size and sphericity.

The model, a polynomial of the second order, was used to evaluate particle size. The trials showed excellent distribution about the linear regression of the model. The Model F test was obtained as 3219.25 for capsule diameter and 253.24 for sphericity, with a *p*-value < 0.05 as the threshold of significance of the parameters and their interaction. The lack of fit F-value for the models was obtained at 0.8006 and 0.20 for microcapsule diameter and sphericity, respectively. The non-significant lack of fit for both models was good, indicating fit models. The ANOVA table distribution is shown in Table 3 and Table 4.

The equations governing the particle diameter and sphericity coefficient determination were given as shown in Equations (4) and (5):

(4) $$\begin{array}{cc} \mathrm{Diameter}(µm)= & 535.082+48.424X_{1}-4.273X_{2}-290.056X_{3}-201.952X_{4}+0.349X_{5}\\ & -16.796X_{1}X_{3}+17.214X_{1}X_{4}+18.575X_{1}X_{5}-14.625X_{2}X_{3}\\ & -12.622X_{2}X_{4}+72.806X_{3}X_{4}-8.576X_{3}X_{5}+15.618X_{4}X_{5}-25.467X_{1}^{2}\\ & +12.962X_{2}^{2}+27.321X_{3}^{2} \end{array}$$

(5) $$\begin{array}{cc} \mathrm{Sphericity}= & 0.821-0.136X_{1}-0.142X_{2}-0.025X_{3}-0.031X_{4}-0.007X_{5}-0.146X_{1}X_{2}\\ & \;+0.021X_{1}X_{3}-0.013X_{1}X_{5}+0.014X_{2}X_{3}+0.011X_{2}X_{4}+0.020X_{2}X_{5}\\ & \;-0.086X_{3}X_{5}+0.0169-0.086X_{1}^{2}-0.048X_{2}^{2}+0.034X_{4}^{2} \end{array}$$

The particle size diameter and sphericity coefficient equations factored all linear parameters, important and possibly important quadratic effects and interrelationships.

#### 3.2.1. Linear Variable Effect on Microcapsule Size and Sphericity

**Figure 6:**
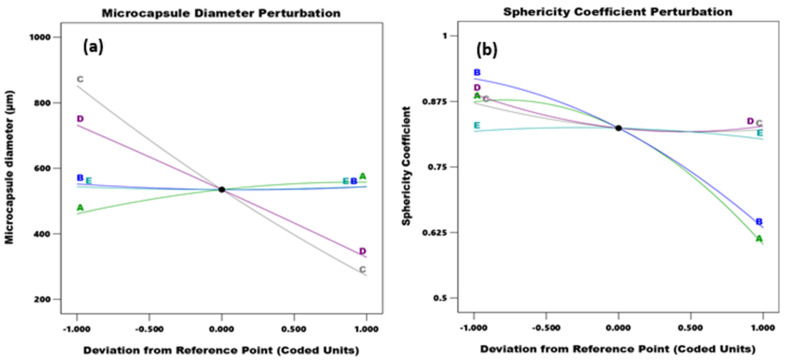
Perturbation summary of independent parameters (A-Alginate concentration; B-Flowrate; C-Needle Size; D-Voltage; E-CaCl_2_ concentration) on (**a**) Microcapsule Diameter and (**b**) Sphericity Coefficient.

The perturbation graphs in Figure 6 depict the linear relationship between the studied independent parameters and their responses. The graph summed up the parameter effect of the independent variables and how they affect the model. With respect to microcapsule diameter, in Figure 6a the biggest contributor to size increment was needle size, followed closely by voltage. A positive deviation from the reference point for voltage and needle size produced smaller microcapsules. A reduction in the needle gauge and voltage decrement resulted in larger particle sizes. Alginate concentration was the next important parameter, an increment of which results in larger microcapsule sizes. However, the contributions of increasing alginate concentration on particle size plateau, and become insignificant at very high alginate concentrations. Flowrate and calcium chloride concentration had the least effect on microcapsule size. While their effect is noticeable, the overall contribution to the model, compared to the other parameters, is small.

The effect of the parameter on the sphericity coefficient is summarized in Figure 6b. Alginate concentration and flowrate were the biggest contributors to sphericity from the model. More spherical capsules were observed with decreasing the flowrate and alginate concentration. Spherical microcapsules can be formed at small and large needle sizes, as shown in Figure 5a,b and Figure 6. The effect of calcium chloride concentration was small. As calcium chloride concentration increases, the graph line begins to taper at the end, explaining that a further increment in calcium chloride concentration could lead to less sphericity. Other parameters could influence the results observed in Figure 4. The increment in voltage leads to less spherical microcapsules.

#### 3.2.2. Effect of Independent Variable Interaction on Microcapsule Particle Size and Sphericity

**Figure 7:**
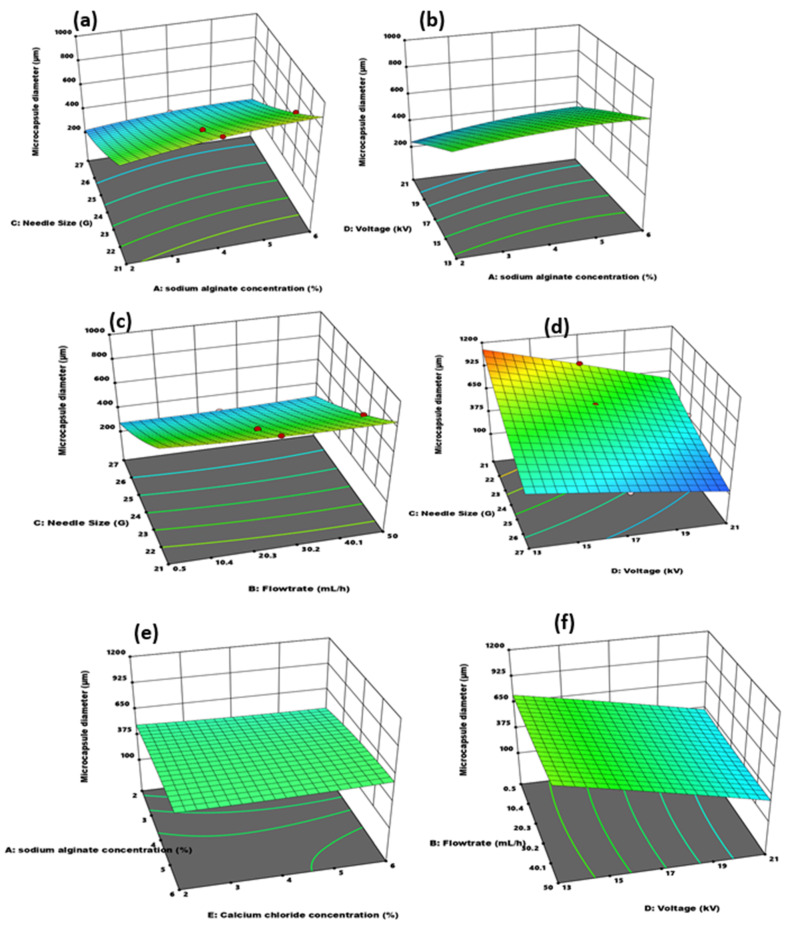
3D Surface plots of varying independent parameter interactions. (**a**) Alginate concentration and needle size; (**b**) voltage and alginate concentration; (**c**) needle size and flowrate; (**d**) voltage and needle size; (**e**) alginate concentration; (**f**) voltage and flowrate and their effect on microcapsule diameter.

**Figure 8:**
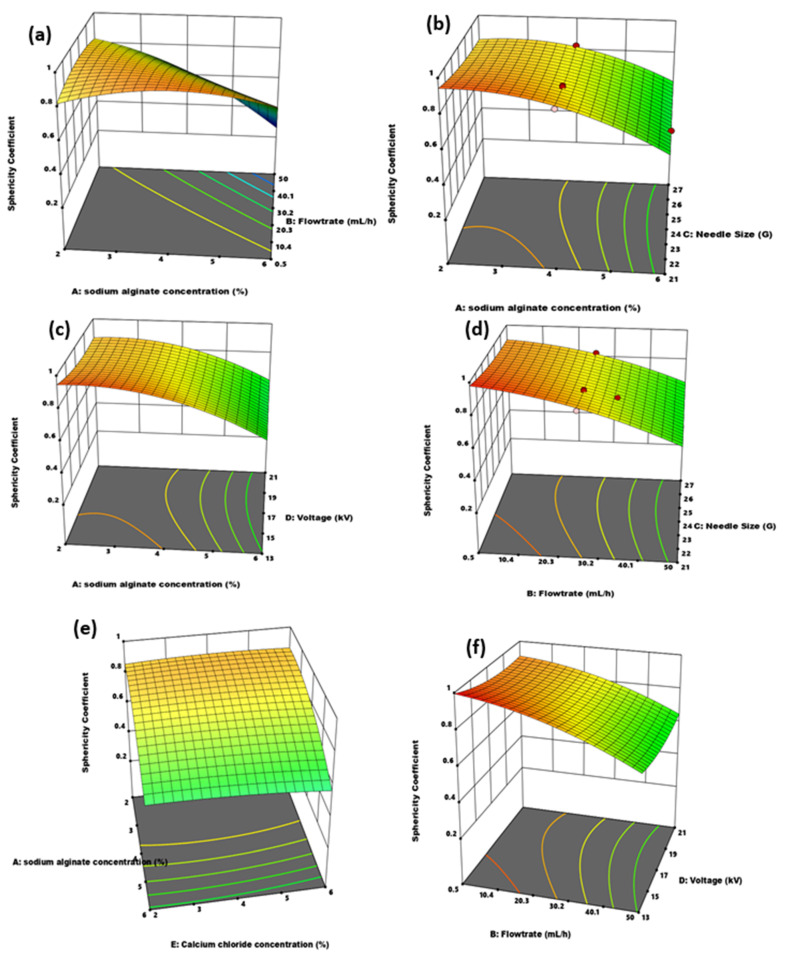
3D surface plot of independent variable interactions. (**a**) Alginate concentration and flowrate; (**b**) alginate concentration and needle size; (**c**) alginate concentration and voltage; (**d**) flowrate and needle size; (**e**) alginate and CaCl_2_ concentration; (**f**) flowrate and voltage and their effect on sphericity.

The effects of the independent variable interaction on the capsule size and sphericity coefficient response parameters are summarized in Figure 7 and Figure 8. In Figure 7a, capsule size decreased with decreasing needle size and alginate concentration. Low viscous alginate solution through a smaller needle diameter favored the formation of smaller microcapsules. Microcapsule diameter increased with increasing sodium alginate concentration, even at a smaller needle diameter. In another investigation [15], a similar trend and effect of viscosity and needle size effect on capsule diameter was observed. Effect on sphericity of the interaction between alginate concentration and needle size was observed in Figure 8b. The more effective parameter was the sodium alginate concentration. Increasing the alginate concentration and pushing through a small-sized needle created elongated and stretched capsules. A third parameter that affects both alginate concentration and needle size is flowrate. Highly viscous alginate solutions electrosprayed through smaller needle diameters have high sphericity if pumped at a very low flowrate [15]. Voltage and alginate concentration effect on diameter is shown in Figure 7b. Microcapsule diameter increased with decreasing alginate concentration and increasing voltage. However, the voltage was the dominant parameter, accounting for the steeper change in microcapsule diameter.

Figure 8c showed sodium alginate concentration to be an important parameter in sphericity determination. The best sphericity was obtained at the lowest voltage and alginate concentration. Low concentrated alginate solutions are quickly broken off by increasing the voltage, leading to smaller and more spherical capsules in the jets electrosprayed into the crosslinker. As the voltage increases, the frequency of the breakage increases until a thin continuous line of fiber formation starts. Thus, an increase in voltage favored small size and less sphericity. The increase in polymer concentration leads to difficulty in breaking the droplet at the tip. The viscous forces prevent a clean break at the tip, leading to tailed-shaped microcapsules resembling tear-, pear- and egg-shaped microcapsules [18], as seen in Figure 3c.

From Figure 7, the flowrate and needle size interaction effects were observed, with respect to capsule diameter. Lower flowrate and smaller needle diameter favored smaller capsule size. The pump rate influences the amount of fluid pushed through the needle diameter, and in effect, the bubble size formed at the tip. A higher flowrate is proportionate to a higher bubble diameter formed at the needle tip, and as such, an overall capsule diameter. However, the dominant parameter was needle size instead of flowrate from the Figure. Observing the sphericity coefficient for flowrate and needle size in Figure 8d, low flowrate and large needle diameter favored better sphericity. The interaction of the two most influential parameters on capsule size was observed in Figure 7d. A steep decrease in size with increasing voltage and decreasing needle diameter showed a strong parameter variation effect. The effect of voltage is felt at the tip of the needle, and as such, the influence of voltage on particle size is on the bubble of the polymer at the nozzle. The droplet size at the tip of the needle is greatly influenced by needle size. If a small bubble-sized polymer is formed at the tip, the effect of voltage is then applied to reduce the size further. The needle size greatly influences the amount of fluid available at the tip for voltage to act on. It can be observed from the Figure that the smallest size was at 21 kV and 27 G, showing that needle size is the more effective of both parameters.

In Figure 7e, calcium chloride and alginate concentrations had minor effects on particle size. The Alginate concentration was the slightly dominant parameter. Figure 7f compared voltage and flowrate on capsule diameter. Smaller size capsules were formed at a low flowrate. However, the effect of flowrate in the presence of voltage was inconsequential, as the major changes in diameter occurred with voltage variation, making it the more important and effective parameter of the two.

Sphericity improves with a lower feed rate and increasing alginate concentration, as shown in Figure 8a. The effect of flowrate at low viscosity is almost negligible on sphericity, with slightly more spherical capsules observed at higher flowrates. The lower sphericity coefficients were recorded at the highest alginate concentration and highest flowrates. In Figure 8e,f, the effect of calcium chloride and sodium alginate concentration and flowrate and voltage on sphericity were observed. In Figure 8e, alginate concentration remained the dominant parameter, and flowrate was the important parameter in Figure 8f. While high voltage is an important parameter for smaller-sized microcapsules, the inverse is true for sphericity, as shown in Figure 8f.

Finally, the numerical optimization of the calcium alginate microcapsules size and sphericity was conducted from the Design-Expert Software. Overall, particle size reduction was given more importance over the sphericity of the microcapsule. The optimum parameter was selected at an alginate concentration of 2.013 *w*/*v*, with a flowrate of 0.560 mL/h, a needle size of 27 G and a 2.024 *w*/*v* calcium chloride concentration. The predicted size was 92.586 µm, with a sphericity coefficient of 0.771, with a 94.54% desirability based on the importance given to the independent variables.

## 4. Conclusions

This study analyzed the electrospraying process and the governing parameters regarding the size and sphericity of the produced calcium alginate microcapsules. The independent process variables were studied, and the interaction between the variables and their correlations to microcapsule diameter and sphericity response were measured. The microcapsule diameter was significantly influenced by the needle size, voltage and alginate concentration. Similar parameter influence was observed regarding sphericity, with flowrate being an additional significant influencing independent parameter. Accordingly, an accurate model for predicting the optimized parameters was successfully developed. Furthermore, variable interactions on response values were also evaluated, showing strong interrelationships on response parameters. As such, OFAT experimental processes are not enough to fully explain the optimization of the electrospray process. Optimized parameters were obtained with respect to the important response parameter, and the optimization was biased towards forming a smaller size with appreciable sphericity.

## Figures and Tables

**Table 1:** Experimental Independent Parameters.

Factor	Name	Units	Coded Low	Coded High
A(X1)	Sodium alginate concentration	%	2.00	6.00
B(X2)	Flowrate	mL/h	0.50	50.00
C(X3)	Needle size	G	21.00	27.00
D(X4)	Voltage	kV	13.00	21.00
E(X5)	Calcium chloride concentration	%	2.00	6.00

**Table 2:** Response Parameters.

Response	Name	Units	Observations	Minimum	Maximum	Mean	Std. Dev.	Ratio
R1	Microcapsule diameter	µm	50.00	75.253	1193.15	523.87	326.21	15.86
R2	sphericity coefficient	N/A	50.00	0.29632	0.9956	0.7731	0.1865	3.36

**Table 3:** ANOVA for Quadratic Model of Microcapsule Diameter.

Source	Sum of Squares	df	Mean Square	F-Value	*p*-Value
Model	5.212 × 10^6^	20	2.606 × 10^5^	3219.25	<0.0001
A(X_1_)-sodium alginate concentration	57,254.67	1	57,254.67	707.30	<0.0001
B(X_2_)-Flowrate	424.16	1	424.16	5.24	0.0295
C(X_3_)-Needle Size	2.153 × 10^6^	1	2.153 × 10^6^	26,602.98	<0.0001
D(X_4_)-Voltage	9.512 × 10^5^	1	9.512 × 10^5^	11,751.21	<0.0001
E(X_5_)-Calcium chloride concentration	3.13	1	3.13	0.0386	0.8456
AB(X_1_X_2_)	320.66	1	320.66	3.96	0.0561
AC(X_1_X_3_)	4144.28	1	4144.28	51.20	<0.0001
AD(X_1_X_4_)	6493.40	1	6493.40	80.22	<0.0001
AE(X_1_X_5_)	4650.78	1	4650.78	57.45	<0.0001
BC(X_2_X_3_)	4288.83	1	4288.83	52.98	<0.0001
BD(X_2_X_4_)	3390.96	1	3390.96	41.89	<0.0001
CD(X_3_X_4_)	76,976.49	1	76,976.49	950.94	<0.0001
CE(X_3_X_5_)	1514.59	1	1514.59	18.71	0.0002
DE(X_4_X_5_)	3414.89	1	3414.89	42.19	<0.0001
$A^{2}(X_{1}^{2})$	1566.25	1	1566.25	19.35	0.0001
$B^{2}(X_{2}^{2})$	647.87	1	647.87	8.00	0.0084
$C^{2}\left(X_{3}^{2}\right)$	1459.96	1	1459.96	18.04	0.0002
Residual	2347.49	29	80.95		
Lack of Fit	1568.52	22	71.30	0.6407	0.8006
Pure Error	778.96	7	111.28		
Cor Total	5.214 × 10^6^	49			

**Table 4:** ANOVA for Quadratic Model of Sphericity coefficient.

Source	Sum of Squares	df	Mean Square	F-Value	*p*-Value
Model	1.69	20	0.0847	253.24	<0.0001
A(X_1_)-sodium alginate concentration	0.4518	1	0.4518	1350.64	<0.0001
B(X_2_)-Flowrate	0.4692	1	0.4692	1402.38	<0.0001
C(X_3_)-Needle Size	0.0165	1	0.0165	49.20	<0.0001
D(X_4_)-Voltage	0.0222	1	0.0222	66.32	<0.0001
E(X_5_)-Calcium chloride concentration	0.0014	1	0.0014	4.28	0.0475
AB(X_1_X_2_)	0.4440	1	0.4440	1327.09	<0.0001
AC(X_1_X_3_)	0.0070	1	0.0070	21.02	<0.0001
AE(X_1_X_5_)	0.0021	1	0.0021	6.36	0.0174
BC(X_2_X_3_)	0.0041	1	0.0041	12.21	0.0015
BD(X_2_X_4_)	0.0027	1	0.0027	8.15	0.0079
BE(X_2_X_5_)	0.0080	1	0.0080	23.95	<0.0001
CE(X_3_X_5_)	0.0072	1	0.0072	21.54	<0.0001
DE(X_4_X_5_)	0.0040	1	0.0040	11.90	0.0017
A^2^(X_1_^2^)	0.0179	1	0.0179	53.42	<0.0001
B^2^(X_2_^2^)	0.0089	1	0.0089	26.60	<0.0001
D^2^(X_4_^2^)	0.0032	1	0.0032	9.57	0.0044
Residual	0.0097	29	0.0003		
Lack of Fit	0.0083	22	0.0004	1.83	0.2093
Pure Error	0.0014	7	0.0002		
Cor Total	1.70	49			

## Data Availability

Not applicable.
